# Effect of domestic cooking on rice protein digestibility

**DOI:** 10.1002/fsn3.884

**Published:** 2019-01-24

**Authors:** Kunlun Liu, Jiabao Zheng, Fusheng Chen

**Affiliations:** ^1^ College of Food Science and Technology Henan University of Technology Zhengzhou China

**Keywords:** amino acids, disulfide bonds, protein body‐I, protein digestibility, rice cooking

## Abstract

The effects of washing, soaking, and common domestic cooking methods (normal cooking, high‐pressure cooking, and microwave cooking) on protein content, in vitro protein digestibility, and amino acid composition of *japonica* and *indica* rice were investigated. All processes in rice domestic cooking did not affect protein content. However, the gastric and gastrointestinal protein digestibilities decreased significantly after cooking. Protein solubility methods were used to observe the formation of disulfide bonds and hydrophobicity interactions after cooking. Disulfide bonds and hydrophobicity interactions were formed during cooking, and the cooking‐induced disulfide bond cross‐linking decreased the protein digestibility observably. Moreover, the solubility of 13 kDa prolamin subunit sharply decreased after cooking due to intramolecular disulfide bond cross‐linking. Therefore, cooking‐induced formation of intramolecular disulfide linkages might stabilize and strengthen the structure of protein body‐I, which exhibited strong resistance to proteases, particularly pepsin. Cooking had limited effect on amino acids.

## 1 INTRODUCTION

Rice (*Oryza sativa* L.) is the predominant staple food and provides over 20% of calorie needs of nearly two‐thirds of the world's population. The protein contents in brown and milled rice are 7.1%–8.3% and 6.3%–7.1%, respectively. With regard to the superior quality of rice protein, the yield of utilizable protein in rice is actually higher than in other cereals (Birla et al., 2017). Rice protein is also usually used in infant diets and elimination diets for people with allergy to food because it is generally recognized as hypoallergenic ingredient.

In some Asian countries, pre‐cooking processes, such as washing and soaking, are performed to remove remaining dust, hull, and bran on the rice kernel surface and improved cooking quality (Yu, Turner, Fitzgerald, Stokes, & Witt, 2017). However, cooking, especially heat‐moisture treatment, can reduce protein digestibility of many cereals, such as rice (Kubota et al., 2014), wheat (Wu, Taylor, Nebl, Ng, & Bennett, 2017), sorghum (Vu, Bean, Hsieh, & Shi, 2017), and millet (Gulati et al., 2017). Factors affecting cereals protein digestibility can be categorized into exogenous and endogenous factors. Exogenous factors mainly include grain organizational structure, starch, and polyphenols. Endogenous factors mainly include secondary structure, hydrophobicity of hydrophobic protein (i.e., kafirins and zeins), and protein cross‐linkings (e.g., disulfide cross‐linking, hydrophobic cross‐linking, and isopeptide cross‐linking). It was reported that, after cooking, the protein digestibilities of brown rice and milled rice decreased by 6.8% and 11.4%, respectively (Bradbury, Collins, & Pyliotis, 1984). The decrease might be due to cooking‐induced formation of a cysteine‐rich core, which can be resistant to proteases rather than isopeptide cross‐linkings. Collier, Barber, and Jna (1998) fed cooked and uncooked rice endosperm protein to mice and sheep, and found that the cooked rice diet produced more fecal protein particles than uncooked rice. In addition, Mujoo, Chandrashekar, and Ali (1998) indicated that disulfide cross‐linkings are formed during rice flaking, and disulfide‐induced aggregation is susceptible to proteolysis. Kubota et al. (2014) found that rice prolamin/protein body‐I (PB‐I) cannot be indigestive in cooked rice. However, Zhang et al. (2010) suggested that the change in rice protein digestibility during cooking was associated with the species of protease. Previous research contributed valuable progresses on cooking‐induced change in rice protein digestibility. However, cooking methods and digestive systems are too diversified. Few studies have systematically investigated changes in protein digestibility and amino acid composition during the entire process of domestic cooking.

This study aimed to (a) evaluate changes in protein content and in vitro digestibility during pre‐cooking processes (washing and soaking) and common domestic cooking; (b) investigate the effects of cooking on amino acid composition of rice; and (c) discuss the causes of changes in protein digestibility upon cooking.

## 2 MATERIALS AND METHODS

### 2.1 Sample preparation

The new harvest rough rice grains of *indica* cultivar T‐You15 (T15) and *japonica* cultivar Xinfeng 2 (X2) were purchased from the Henan Academy of Agricultural Science in November 2016. Milled rice samples were obtained by removing husks and milling with a rice dehulling machine to 9%.

### 2.2 Cooking

Rice cooking methods (normal cooking, high‐pressure cooking, and microwave cooking) were performed by our previous study (Liu, Zheng, & Chen, 2018), which based on Chinese national standard and common cooking methods in Asia. In addition to above cooking operations, rice samples were also cooked in reducing agent (0.1 M 2‐mercaptoethanol) and ionic/nonionic detergents (2% Triton X‐100 and 2% CHAPS), respectively, in order to explore any effect of disulfide bonds or hydrophobic interactions during rice cooking on protein digestibility. The cooked samples were freeze‐dried and stored for further analysis.

### 2.3 Determination of in vitro protein digestibility

The gastric protein digestibility (G‐PD) and gastrointestinal protein digestibility (GI‐PD) were measured as the method described by Deng, Luo, Wang, and Zhao (2015). In the simulated gastric procedure, 1 g of rice sample was mixed with 17 ml of HCl (0.1 M) and 2.5 ml of pepsin solution (2.5 mg/ml in 0.1 M HCl). The mixture was blended using a vortex mixer for 2 min and shook in a digital water bath oscillator for 2 hr at 37°C. In the simulated gastrointestinal procedure, which was based on simulated gastric digestion, 0.5 M NaOH was added to adjust the pH of the mixture to 8.0. And 2.5 ml of pancreatic solution [2.5 mg/ml in 0.1 M phosphate buffer (pH 8.0)] was added to initiate the 2‐hr gastrointestinal digestion at 37°C. Then, 10 ml of 100 g/L trichloroacetic acid was added to stop gastrointestinal digestion. The mixture was centrifuged at 9,000 × *g* for 15 min, and 10 ml of supernatant was collected for Kjeldahl measurement. The protein digestibility was calculated as the protein in supernatant to the protein in samples. The total protein content was measured by Automatic Kjeldahl apparatus in accordance with AACC method 46‐13 (AACC, 2000).

### 2.4 Determination of changes in protein interactions

The protein interactions during rice cooking were evaluated by protein solubility methods by using different solvent systems described by Liu and Hsieh (2008). The selective reagents were a combination of five extracting solutions as follows: (a) 0.1 M phosphate buffer + 8 M urea + 0.05 M dithiothreitol (DTT) + 2 M thiourea + 2% Triton X‐100 + 2% CHAPS; (b) 0.1 M phosphate buffer + 8 M urea + 0.05 M DTT; (c) 0.1 M phosphate buffer + 0.05 M DTT + 2 M thiourea + 2% Triton X‐100 + 2% CHAPS; (d) 0.1 M phosphate buffer + 8 M urea + 2 M thiourea + 2% Triton X‐100 + 2% CHAPS; and (e) 0.1 M phosphate buffer.

Each rice sample was homogenized with 10 ml extracting solution by for 2 min and shaked for 2 hr at 25°C. Then, the homogenates were centrifuged for 20 min at 10,000 × *g*. The protein solubility was calculated as the protein in supernatant to the protein in samples. Each measurement was performed in triplicate.

### 2.5 Reducing and nonreducing SDS‐PAGE

Sodium dodecyl sulfate polyacrylamide gel electrophoresis (SDS‐PAGE) was carried out using the discontinuous system (12% separating/5% stacking gel) according to the method of Liu, Chen, Yan, Gu, and Yang (2011). Isoelectric focusing (IEF) buffer and the reagent without DTT were using to extract rice protein in reduced and nonreduced condition, respectively.

### 2.6 Amino acid composition analysis

Amino acid composition was measured with an amino acid analyzer (S433D, Sykam, Germany) according to Liu, Zheng, and Chen (2017). Rice flour (equal to 10–20 mg protein) was proteolyzed with 10 ml hydrochloric acid (6 M) and 3–4 drops of phenol.

### 2.7 Statistical analysis

Values were expressed as means ± standard deviations. The significant difference was determined at the *p *<* *0.05 level for Duncan's multiple range test by using SPSS software (version 20.0, Chicago, USA).

## 3 RESULTS AND DISCUSSION

### 3.1 Effect of cooking on rice protein content and in vitro digestibility

Table 1 shows the protein contents in X2 and T15 subjected to various pre‐cooking (washing and soaking) and cooking. The protein contents showed no significant (*p *>* *0.05) changes during cooking. Zhang et al. (2010) also reported no significant change was observed in protein content of cooked milled rice. Although some soluble proteins were dissolved in water during initial cooking period, the water can be absorbed by rice during cooking.

**Table 1:** Protein content of raw rice (RR), washed rice (WR), soaked rice (SR), normally cooked rice (NR), high‐pressure cooked rice (HR), and microwave cooked rice (MR)

Samples	Protein content (%)	
	X2	T15
RR	8.11 ± 0.06^a^	8.58 ± 0.06^a^
WR	8.02 ± 0.02^a^	8.48 ± 0.03^a^
SR	8.08 ± 0.07^a^	8.45 ± 0.02^a^
NR	8.18 ± 0.14^a^	8.50 ± 0.13^a^
HR	8.17 ± 0.13^a^	8.44 ± 0.09^a^
MR	8.11 ± 0.04^a^	8.50 ± 0.12^a^

Note

Means followed by different small cases for the same column are significantly different (*p *<* *0.05).

**Figure 1:**
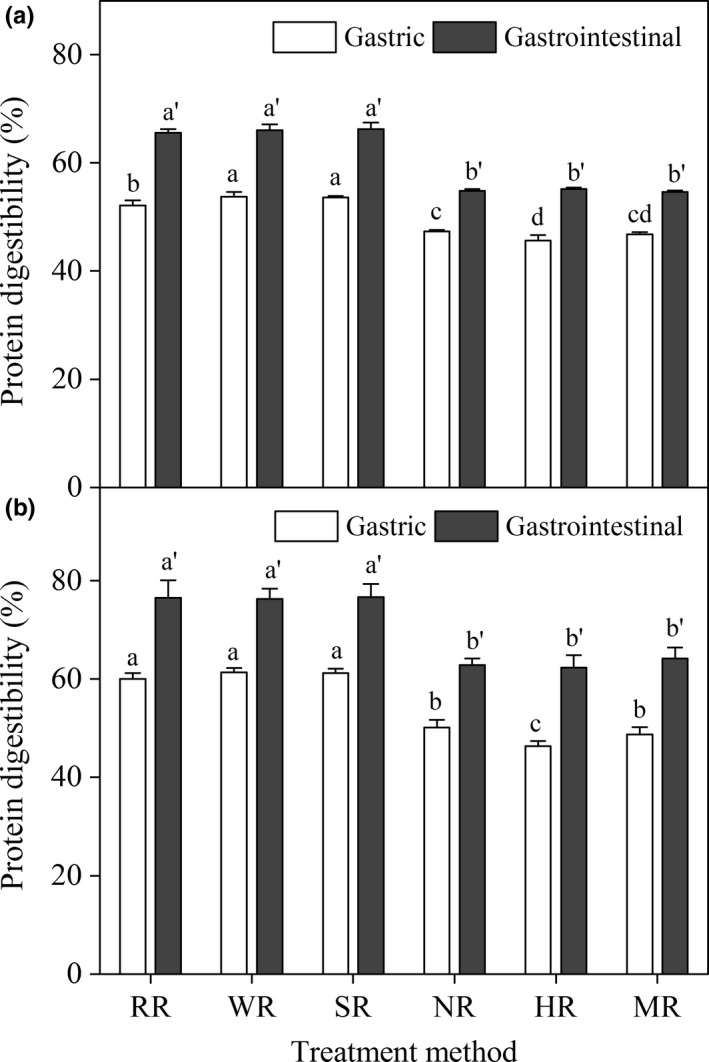
Effect of cooking on protein digestibility of X2 (a) and T15 (b). Abbreviations RR, WR, SR, NR, HR, and MR represent raw rice, washed rice, soaked rice, normally cooked rice, high‐pressure cooked rice, and microwave cooked rice, respectively. Different lowercase letters were used to show significant differences in gastric and gastrointestinal protein digestibilities between rice samples at various treatments (*p *<* *0.05)

Figure 1 shows protein digestibility of X2 and T15. No significant change was observed in the G‐PD and GI‐PD of T15, and the GI‐PD of X2 after pre‐cooking. However, G‐PD of X2 increased by 3.1% after washing. In general, water migration in the rice kernel was closely related to the soaking temperature. Thus, the limited effect of pre‐cooking on protein digestibility might be due to low water migration at 25°C (Tong et al., 2017).

The protein digestibility significantly decreased after cooking (Figure 1). For example, compared with protein digestibility in raw rice, the G‐PD of X2 decreased by 9.4%, 12.6%, and 10.3%, and the GI‐PD of X2 decreased by 16.5%, 17.1%, and 16.8%, subjected to normal cooking, high‐pressure cooking, and microwave cooking, respectively. These results suggested that cooking methods can affect protein digestibility significantly. Bradbury et al. (1984) found that the GI‐PD of brown rice and milled rice decreased from 73% to 68% and from 79% to 70%, respectively. The higher decline in protein digestibility in the present study might be due to the differences in cooking methods and rice varieties. In addition, Zhang et al. (2010) reported no significant change in rice protein digestibility was observed after cooking in three‐enzyme assay (trypsin, chymotrypsin, and aminopeptidase). However, in four‐enzyme assay with bacterial protease added, the protein digestibility significantly increased after cooking. The opposite results indicated that cooking‐induced change in protein digestibility was related to the type of protease. Thus, protein digestibility reduction was mainly attributed to the accessibility of the specific peptide bonds to the pepsin being impeded.

### 3.2 Analysis of cooking‐induced rice protein interactions

**Figure 2:**
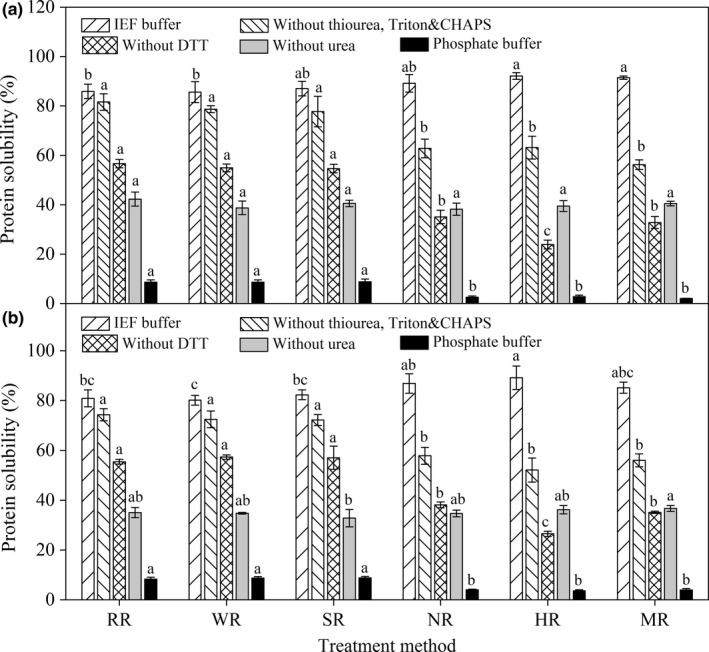
Effect of cooking on protein solubility of X2 (a) and T15 (b). Abbreviations RR, WR, SR, NR, HR, and MR represent raw rice, washed rice, soaked rice, normally cooked rice, high‐pressure cooked rice, and microwave cooked rice, respectively. Different lowercase letters were used to show significant differences in gastric and gastrointestinal protein digestibilities between rice samples at various treatments (*p *<* *0.05)

The protein solubility of X2 and T15 in phosphate buffer significantly decreased after cooking (Figure 2). This finding was due to heat‐induced rice protein denaturation, which could induce partial unfolding and association/aggregation of protein and decrease its solubility. In general, the protein solubility of X2 and T15 in IEF buffer was significantly higher than that in other four solvent systems (Figure 2). It indicated that hydrogen bonds, hydrophobic interactions, and disulfide bonds were all responsible for the rice protein structure. However, heat‐induced unfolding of rice protein and the crack of the starch structure could facilitate rice protein dissolution in IEF buffer. This condition might result in the significant increase in rice protein solubility in IEF buffer after cooking. The protein solubilities of X2 and T15 in the two solvent systems, which subtracted DTT, and removed thiourea, Triton and CHAPS, both significantly decreased after cooking. Therefore, it showed heat‐induced formation of hydrophobic interactions and disulfide bonds. This condition might prove the hypothesis of Kubota et al. (2014), who suggested that cooking might lead to hydrophobic interactions and disulfide linkages in PB‐Is. Rice protein solubility in the solvent without urea was almost constant during cooking processes, suggested that changes of hydrogen bonds did not occur during cooking‐induced protein interactions despite its important role in the native structure of rice protein.

In addition, the protein solubilities of high‐pressure cooked rices (both X2 and T15) were significantly lower compared with those subjected to the other cooking methods in the reagent subtracted DTT (Figure 2). It revealed that high‐pressure cooking might lead to additional disulfide bonds cross‐linking caused by temperature increase.

### 3.3 Effect of reducing agent and ionic/nonionic detergents on in vitro protein digestibility

**Figure 3:**
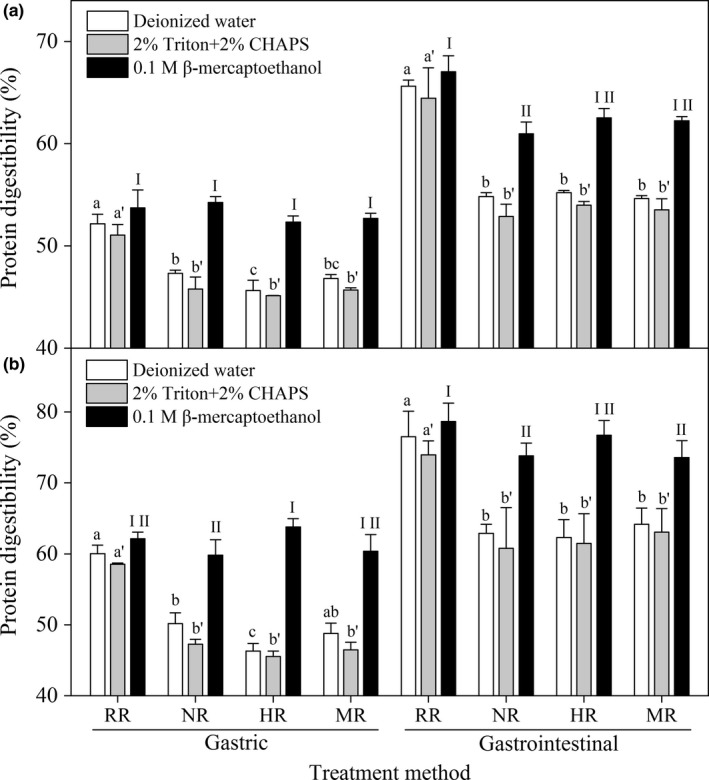
Effect of reducing agents and ionic/nonionic detergents on protein digestibility of X2 (a) and T15 (b) with various treatments. Abbreviations RR, WR, SR, NR, HR, and MR represent raw rice, washed rice, soaked rice, normally cooked rice, high‐pressure cooked rice, and microwave cooked rice, respectively. Different lowercase letters and Roman number were used to show significant differences in gastric and gastrointestinal protein digestibilities between rice samples at various treatments (*p *<* *0.05)

Both hydrophobic interactions and disulfide linkages were involved in cooking‐induced protein cross‐linking. However, how they affected protein digestibility was not clear. Thus, X2 and T15 were cooked in the presence of β‐mercaptoethanol or Triton and CHAPS. As shown in Figure 3, the G‐PD and GI‐PD of X2 and T15 cooked in the presence of Triton and CHAPS were slightly lower than those of the rice cooked by deionized water. Moreover, the G‐PD and GI‐PD significantly decreased after cooking in the presence of Triton and CHAPS compared with those of the raw rice. The results suggested that the decline in rice protein digestibility might be not significantly affected by the cooking‐induced hydrophobic interactions, and even the hydrophobic interactions in native structure. In addition, the presence of Triton and CHAPS could adversely affect the structures of protease, especially pepsin.

It was found that the digestibility of raw rice protein increased by 3.5% after soaking in β‐mercaptoethanol (Hamaker, Kirleis, Butler, Axtell, & Mertz, 1987). However, in the four‐enzyme digestive system consisting of trypsin, chymotrypsin, and aminopeptidase, the addition of reducing agents inhibited the multi‐enzyme digestibility of uncooked rice (Zhang et al., 2010). In the present study, no significant changes were observed in the G‐PD and GI‐PD of raw rice after soaking in 0.1 M β‐mercaptoethanol, despite their slight increase by 2.1%–3.5%. However, the G‐PD of the rice cooked with β‐mercaptoethanol was significantly higher than that of rice cooked by deionized water. No significant difference was observed between the G‐PD of β‐mercaptoethanol cooked rice and β‐mercaptoethanol soaked rice. This result suggested that β‐mercaptoethanol could increase the G‐PD of cooked rice to the level of raw rice soaked in the presence of β‐mercaptoethanol. Similar results were found in the GI‐PD of β‐mercaptoethanol cooked rice. However, the GI‐PD of β‐mercaptoethanol cooked rice was significantly higher than that of the rice cooked by deionized water. These findings indicated that the formation of disulfide bond cross‐linking during cooking negatively affected the rice protein digestibility, whereas disulfide bonds in the native structure might have a limited effect on protein digestibility. Meanwhile, cooking‐induced disulfide bond cross‐linking could mainly inhibit pepsin digestion. Hence, the rice protein digestibility did not decrease in the multi‐enzyme digestive system, excluding pepsin, in the study of Zhang et al. (2010). Kubota et al. (2014) assumed that rice PB‐I structure can be strengthened as a result of hydrophobicity interactions and disulfide bonds during cooking, possibly reducing rice protein solubility. However, in this study, cooking‐induced formation of disulfide bonds reduced rice protein digestibility, whereas similar result was not detected in hydrophobicity interactions occurring during cooking.

### 3.4 Analysis of rice protein profile during cooking

**Figure 4:**
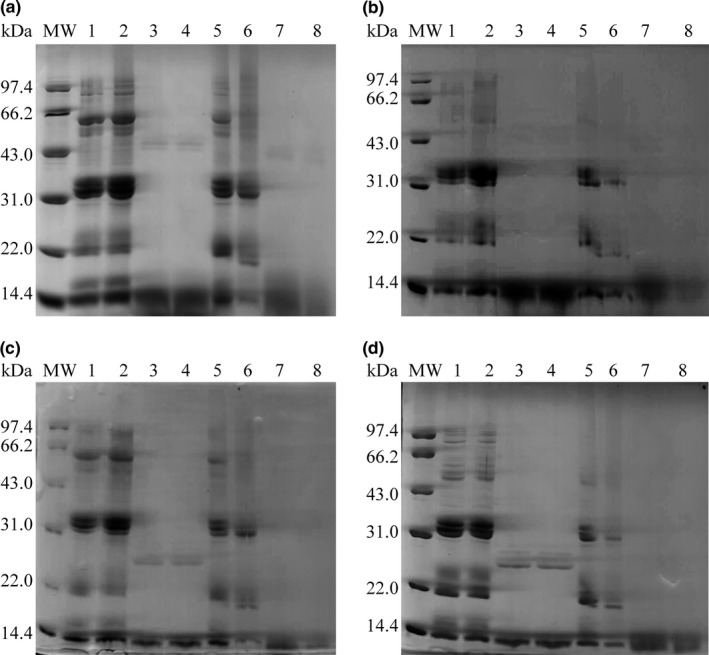
SDS‐PAGE patterns of rice protein in X2 and T15. (a) and (b) represent gastric phase of X2 and T15, respectively; C and D represent gastrointestinal phase of X2 and T15, respectively. MW: molecular weight markers; 1: uncooked X2 before digestion (reduced condition); 2: cooked X2 before digestion (reduced condition); 3: uncooked X2 after digestion (reduced condition); 4: cooked X2 after digestion (reduced condition); 5: uncooked X2 before digestion (nonreduced condition); 6: cooked X2 before digestion (nonreduced condition); 7: uncooked X2 after digestion (nonreduced condition); 8: cooked X2 after digestion (nonreduced condition)

In order to further confirm the relationship between cooking, protein digestibility, and disulfide cross‐linking, the high‐pressure cooked rice protein profiles were analyzed via SDS‐PAGE (Figure 4). High‐pressure cooked rice was selected because of its strong disulfide linkages and low protein digestibility. Rice glutelin was initially found in polypeptide subunits, called glutelin precursor, with molecular weights (MWs) of 51–57 kDa (Yamagata, Sugimoto, Tanaka, & Kasai, 1982). Generally, the glutelin precursor can be hydrolyzed into α‐glutelin and β‐glutelin within the range of 30–40 and 19–23 kDa, respectively (Agboola, Ng, & Mills, 2005; Amagliani, O'Regan, Kelly, & O'Mahony, 2017). Glutelin precursor was observed in this study despite its difference shown in the polypeptide subunits of X2 and T15. The differences might be associated with rice variety and maturity. The polypeptide subunits of α‐glutelin and β‐glutelin were in range of 32–35 and 19–21 kDa in X2 and T15, respectively. Furthermore, consistent with the results of Hibino et al. (1989) and Ogawa et al. (1987), prolamin polypeptide subunits with MWs of 16 and 13 kDa were found, and the 13 kDa subunit was predominant.

As shown in Figure 4a and b, the lane 2 intensity was slightly higher than that of lane 1. This finding might be due to the destruction of starch structure and protein denaturation, which could facilitate rice protein dissolution in IEF buffer, after cooking. These results were in line with our observation, suggesting that protein solubility of cooked rice was slightly higher than that of raw rice in IEF buffer. By contrast, in nonreduced conditions, the intensity of polypeptide subunits in cooked rice protein was lower than that in raw rice protein, and no new polypeptide subunit was found. This finding indicated that intramolecular disulfide bond cross‐linking was involved in heat‐induced rice protein interactions rather than in intermolecular protein cross‐linking, possibly resulting in the cooking‐induced reduction of protein solubility with removal of the reducing reagent.

Under reduced conditions (lanes 3 and 4 in Figure 4a and b), except for the polypeptide subunits of prolamin with MW of 13 kDa, pepsin hydrolyzed all of other rice protein polypeptide subunits. Xia et al. (2012) reported that polypeptide subunits, including glutelin precursor, α‐glutelin, β‐glutelin, and α‐globulin, can be hydrolyzed in vitro by gastric digestion. By contrast, prolamin polypeptide subunits with MW of 16 kDa were not digested, possibly due to PB‐I, which could resist attacks of pepsin and trypsin when its structure was not destroyed by alkali. Kubota et al. (2014) also reported that rice prolamin indigestibility was associated with PB‐I, which could be degraded or weakened by alkali, and thus increased protein digestibility of rice prolamin (Kubota et al., 2010). Therefore, in the present study, the hydrolysis of the 16 kDa prolamin might be due to the IEF buffer corrosion on PB‐I structure.

In addition, decrease of intensity was observed in 13 kDa prolamin after cooking in nonreducing conditions (lanes 5 and 6 in Figure 4a and b). These results indicated that, despite its poor digestibility, the solubility of 13 kDa prolamin still sharply decreased after cooking due to intramolecular disulfide bond cross‐linking. This phenomenon might be closely related to the reduction of rice protein digestibility during cooking. Similar results were also found in lanes 3, 4, 7, and 8 in Figure 4c and d, suggesting that the 13 kDa subunit was still indigestible after further in vitro intestinal digestion. Higher GI‐PD than G‐PD was mainly caused by the low MW of digested rice protein that underwent in vitro gastrointestinal digestion.

### 3.5 Effect of cooking on amino acid composition

As shown in Table 2, the total amino acids in raw T15 were slightly higher than that in raw X2 due to the high protein content in the former (Table 1). For detected essential amino acids (EAA), significant changes were only observed in high‐pressure cooked T15, which indicated that the effect of cooking on rice EAA was limited. Ilo and Berghofer (2003) found that some thermal instability amino acids including lysine, arginine, cysteine, methionine, and tryptophan are lost during oat powder extrusion. By contrast, no significant change was observed in these amino acids except cysteine. This finding may because the intensity of rice cooking was milder than extrusion. However, slight reduction was observed in cysteine of X2. It may due to cooking‐induced oxidation in cysteine and/or additional loss of cysteine during acid hydrolysis. In addition, decreases of serine and proline occurred after cooking, especially at high‐pressure cooking and microwave cooking, despite that microwave cooking is regarded as a preferable method that can prevent the excessive loss of nutrients in food matrix during cooking.

**Table 2:** Amino acid composition of rice with various treatments (mg/g)

Amino acid	X2				T15			
	RR	NR	HR	MR	RR	NR	HR	MR
Asp	3.91 ± 0.11^a^	3.80 ± 0.08^a^	3.81 ± 0.05^a^	3.87 ± 0.07^a^	3.57 ± 0.04^a^	3.75 ± 0.25^a^	3.66 ± 0.04^a^	3.59 ± 0.01^a^
Thr	1.13 ± 0.02^a^	1.12 ± 0.10^a^	1.10 ± 0.04^a^	1.05 ± 0.06^a^	1.20 ± 0.05^a^	1.16 ± 0.07^a^	1.21 ± 0.07^a^	1.07 ± 0.18^a^
Ser	3.33 ± 0.10^a^	3.09 ± 0.58^a^	2.96 ± 0.45^a^	3.12 ± 0.32^a^	3.13 ± 0.03^a^	3.15 ± 0.02^a^	2.67 ± 0.21^b^	2.71 ± 0.14^b^
Glu	14.31 ± 0.31^a^	14.15 ± 0.04^a^	14.15 ± 0.19^a^	14.02 ± 0.10^a^	13.89 ± 0.11^a^	13.78 ± 0.11^a^	14.58 ± 0.49^a^	13.29 ± 0.90^a^
Gly	2.68 ± 0.01^a^	2.69 ± 0.01^a^	2.68 ± 0.02^a^	2.67 ± 0.05^a^	2.71 ± 0.03^a^	2.70 ± 0.02^a^	2.83 ± 0.03^a^	2.67 ± 0.32^a^
Ala	3.77 ± 0.04^a^	3.86 ± 0.02^a^	3.71 ± 0.18^a^	3.74 ± 0.02^a^	3.87 ± 0.02^a^	3.83 ± 0.08^a^	3.61 ± 0.03^a^	3.61 ± 0.03^a^
Val	3.81 ± 0.01^a^	3.96 ± 0.08^a^	3.81 ± 0.22^a^	3.84 ± 0.13^a^	3.93 ± 0.01^a^	3.80 ± 0.11^a^	3.88 ± 0.06^a^	3.78 ± 0.08^a^
Met	0.90 ± 0.16^a^	0.98 ± 0.12^a^	1.09 ± 0.28^a^	1.06 ± 0.26^a^	1.37 ± 0.09^a^	1.21 ± 0.07^a^	1.46 ± 0.15^a^	1.35 ± 0.09^a^
Ile	3.31 ± 0.05^a^	3.31 ± 0.45^a^	3.37 ± 0.23^a^	3.35 ± 0.03^a^	3.55 ± 0.03^ab^	3.71 ± 0.18^a^	3.35 ± 0.12^b^	3.62 ± 0.12^ab^
Leu	6.42 ± 0.17^a^	6.38 ± 0.45^a^	6.44 ± 0.15^a^	6.45 ± 0.02^a^	6.24 ± 0.01^a^	6.13 ± 0.07^a^	6.38 ± 0.64^a^	6.75 ± 0.06^a^
Tyr	0.87 ± 0.10^a^	0.96 ± 0.04^a^	1.18 ± 0.35^a^	1.23 ± 0.36^a^	1.12 ± 0.09^a^	1.08 ± 0.04^a^	1.01 ± 0.06^a^	1.01 ± 0.06^a^
Phe	3.77 ± 0.20^a^	3.77 ± 0.04^a^	3.75 ± 0.01^a^	3.73 ± 0.01^a^	3.72 ± 0.11^a^	3.72 ± 0.07^a^	3.38 ± 0.36^a^	3.64 ± 0.28^a^
His	2.01 ± 0.13^a^	2.06 ± 0.14^a^	2.12 ± 0.02^a^	2.11 ± 0.02^a^	2.20 ± 0.05^a^	2.29 ± 0.11^a^	2.39 ± 0.12^a^	2.56 ± 0.22^a^
Lys	2.80 ± 0.30^a^	2.83 ± 0.06^a^	2.80 ± 0.06^a^	2.81 ± 0.13^a^	3.14 ± 0.07^a^	3.07 ± 0.04^a^	2.83 ± 0.07^a^	3.24 ± 0.33^a^
Arg	5.38 ± 0.08^b^	5.71 ± 0.11^ab^	5.77 ± 0.08^a^	5.81 ± 0.20^a^	5.64 ± 0.04^a^	5.63 ± 0.01^a^	5.70 ± 0.09^a^	5.81 ± 0.22^a^
Pro	3.88 ± 0.74^a^	2.98 ± 0.17^a^	2.78 ± 0.34^a^	2.81 ± 0.22^a^	3.74 ± 0.08^a^	3.56 ± 0.40^ab^	2.99 ± 0.12^bc^	2.67 ± 0.1^c^
Cys	0.23 ± 0.00^a^	0.14 ± 0.05^b^	0.17 ± 0.04^ab^	0.18 ± 0.05^ab^	0.023 ± 0.01^a^	0.19 ± 0.02^a^	0.20 ± 0.04^a^	0.17 ± 0.01^a^
TAA	62.50	61.81	61.69	61.85	63.26	62.76	62.15	61.53
EAA	22.14	22.35	22.36	22.29	23.15	22.80	22.49	23.45
E/T (%)	35.4	36.2	36.2	36.0	36.6	36.3	36.2	38.1

Notes

EAA: essential amino acids; TAA: total amino acids.

E/T (%) = EAA/TAA.

Means followed by different small cases for the same amino acid and the same variety are significantly different (*p *<* *0.05).

## 4 CONCLUSIONS

The processes in rice domestic cooking, including washing, soaking, normal cooking, high‐pressure cooking, and microwave cooking, showed no significant effects on protein content. However, both G‐PD and GI‐PD significantly decreased after cooking, despite the limited effect of rice pre‐cooking on protein digestibility. Disulfide bonds and hydrophobicity interactions were formed during the three cooking. However, cooking‐induced hydrophobicity interactions might not affect the protein digestibility. By contrast, disulfide bond cross‐linking during cooking decreased the protein digestibility observably. The heat‐induced formation of intramolecular disulfide linkages during cooking might stabilize and strengthen rice PB‐I, showing stronger resistance to protease, especially pepsin. In general, cooking had limited effects on rice amino acids.

## CONFLICT OF INTEREST

The authors declare that they have no conflict of interests.

## ETHICAL REVIEW

This study does not involve any human or animal testing.
